# Reproducibility of image-based computational models of intracranial aneurysm; methodological issue

**DOI:** 10.1186/s12938-016-0223-9

**Published:** 2016-09-16

**Authors:** Siamak Sabour, Zhi-Yong Li

**Affiliations:** 1 Safety Promotion and Injury Prevention Research Center, Shahid Beheshti University of Medical Science, Tehran, I.R. Iran; 2 Department of Clinical Epidemiology, School of Health, Shahid Beheshti University of Medical Sciences, Tehran, I.R. Iran; 3 Biomechanics Laboratory, School of Biological Science and Medical Engineering, Southeast University, Nanjing, 210096 P.R. China; 4 School of Chemistry, Physics and Mechanical Engineering, Queensland University of Technology, Brisbane, QLD 4001, Australia

**Keywords:** Aneurysm, Angiography, CFD, CTA, MRA, Reproducibility, Methodology, Misdiagnosis, Mismanagement

I was interested to read the paper by Ren and colleagues published in May 2016 issue of *Biomed Eng Online* [1]. The authors aimed to evaluate the reproducibility of computational models of intracranial aneurysm reconstructed from computed tomography angiography (CTA), magnetic resonance angiography (MRA) and 3D rotational angiography (3DRA) [1, 2].

As the authors mentioned, model geometry and hemodynamic parameters were compared between the three models. They reported that in respect of hemodynamic parameters, all three models showed a similar distribution: low average WSS at the sack, high OSI at the body and high average WSSG at the neck. However, there was a large variation in the average WSS (Δ = 34 ± 5.13 % for CM, Δ = 40.6 ± 9.21 % for MM) [1, 2].

Regarding reliability, it is crucial to know that an individual based approach instead of group based (average) should be considered [3–7]. The reason is in reliability assessment; we should consider individual results and not global average. Therefore, intra class correlation coefficient (ICCC) single measure instead of average measure should be reported to correctly assess the reliability. In other words, possibility of getting exactly the same average of a variable between three models with no reliability at all is high [3–7].

Moreover, reporting significant difference between two methods is completely different methodological issue from clinically importance of the mentioned difference. In reliability analysis, depending on sample size, a negligible clinical difference can be statistically significant [3–7].

As the authors pointed out in their conclusion, CTA and MRA have no significant differences in reproducing intracranial aneurysm geometry. There might be some significant differences in hemodynamic parameters between the three imaging-based models and this need to be considered when interpreting the CFD results of different imaging-based models. Such a conclusion can be a misleading message and should be avoided by researcher otherwise; misdiagnosis and mismanagement of the patients cannot be avoided.

## Reply to Letter by Dr. Sabour

### Yuan Ren, Guo-Zhong Chen, Zhen Liu, Yan Cai, Guang-Ming Lu, Zhi-Yong Li

We thank Dr. Sabour for his interest in our recent study [1, 2], which gives our opportunity to discuss and clarify our results further.

We would like firstly to point out that our results should in no way be used as guidance for clinical decision-making or patient management for three different imaging modalities. In our study, no significant differences in both morphological and hemodynamic parameters were detected. Only one suggestion was made in terms of the interpretation of the computational fluid dynamics (CFD) results because some variation of average wall shear stress (WSS) was found between the three patient-specific models based on different angiographies.

In this study, we found a similar distribution in hemodynamic parameters in three types of CFD models developed from three different types of angiographies. The difference of each parameter was calculated as: (|CM (or MM) − DM|)/DM × 100 %. The means and standard errors of all morphological and hemodynamic parameters were calculated. The differences between DM and CM or DM and MM were analyzed by a paired nonparametric Wilcoxon test. This study was designed to detect any difference in the simulation results rather than to test the reliability of different imaging methods or measurements. Therefore we have not used intra-class correlation coefficient (ICC). We have only taken DSA as a golden standard and compared the difference in geometrical and CFD parameters from models reconstructed from CTA, MRA and DSA.

In the current literature, there are many different types of patient-specific CFD models that are developed from different types of clinical imaging. Therefore, this study was focused to investigate whether these CFD results are comparable or not. There were no significant differences between the commonly used morphological and CFD parameters between the three different models. Some variation in WSS was found although it was not significant and therefore we suggest that we need to be careful when interpreting the simulation results in the future. This study was not aimed to compare any clinically used parameters and it should not be taken to influence any clinical-decision making.
